# Hall Technique for Carious Primary Molars: A Review of the Literature

**DOI:** 10.3390/dj8010011

**Published:** 2020-01-17

**Authors:** Doua H. Altoukhi, Azza A. El-Housseiny

**Affiliations:** 1 Pediatric Dentistry Department, Faculty of Dentistry, King Abdulaziz University, Jeddah 21589, Saudi Arabia; dr.doaa.t@icloud.com; 2 Pediatric Dentistry Department, Faculty of Dentistry, Alexandria University, Alexandria 21526, Egypt

**Keywords:** Hall technique, use, primary molars, carious, pediatric dentistry

## Abstract

The high frequency of caries in primary teeth and its inadequate treatment are major public health problems during childhood. Nowadays, the Hall technique is one of the methods used for biological sealing in carious lesions in primary molars. Thus, the bacteria will be sealed from oral environment and the caries will be inactive. The objective of this article was to provide an updated search on the Hall technique description, indication, contraindication, advantages, concerns, success and failure, cost-effectiveness, acceptability, and preference in pediatric dentistry, and to compare the Hall technique with traditional crown preparation and conventional treatment options for carious primary molars. A discussion of the recently published articles on the Hall technique reveals that the Hall technique is considered a promising restorative option with high acceptability and longevity; with low failure rate for managing carious primary molars compared to conventional treatment modalities used in primary care settings. Furthermore, the survival rate of stainless steel crowns (SSCs) is considered high, whether provided using Hall technique or traditional preparation by a pediatric dentist. Thus, the Hall technique can be an effective addition to the clinician’s range of treatment options for carious primary molars. However, it should be chosen in restricted cases.

## 1. Introduction

Dental caries in primary teeth is considered as the most common oral disease of childhood and has been studied in different countries worldwide [1]. The prevalence rate of early childhood caries (ECC) is between 1% and 12% in most developed countries [2]. However, the prevalence is higher in less developed countries, reaching 70% [3]. 

Traditional methods of managing carious primary molars in children include restoration with amalgam, composite resin, compomer, glass ionomer, and stainless steel crowns (SSCs) using conventional tooth preparation [4] or extraction [5]. Recently, silver diamine fluoride (SDF) is being used as a non-invasive treatment option [6].

Most of the methods for managing carious primary molars are done in secondary care settings or by specialists in private clinics. Restorations carried out by general dental practitioners (GDPs) showed less promising results [7]. The high frequency of caries in primary teeth, and its inadequate treatment, is considered a major public health problem during childhood and could significantly affect children’s lives. Fifteen percent of Scottish children have had at least one extracted tooth by the age of five years [8]. By the age of eight years old, this percentage is increased to 42% [9]. Furthermore, many children have to accept dental pain; with approximately half of children with carious primary teeth stated to have attended the general dental practitioners’ clinics with dental pain [10]. Despite the fact that preformed metal crowns (PMCs) are recommended as the optimum treatment for managing primary molars where caries includes two or more surfaces [11], they are not widely used by the primary care dentists due to the difficulties in the technique and in the ability of children to accept invasive treatment that involves local anesthesia and tooth preparation [12].

The Hall technique is one of the methods used nowadays for sealing in caries in primary molars [13]. The Hall technique using preformed metal crowns (PMCs) was first introduced in the literature in 2006 by Dr. Norna Hall, a general dentist from Scotland. Using the Hall technique, the crown is placed without local anesthesia, caries removal, or tooth preparation. An appropriate size of PMC should be chosen and filled with glass ionomer cement. Then, the crown is fitted over the carious primary molar by either the dentist’s finger pressure, or the child’s biting force [14]. 

The Hall technique has very straightforward biological principles. It can arrest caries and protect the primary tooth until shedding. By using the Hall technique, the superficial plaque layer, which is the most essential layer in the biofilm for caries progression, is left and sealed along with the carious lesion. As a response, the plaque biofilm composition will be changed to a less cariogenic flora. Therefore, this technique may arrest or at least slows down caries progression in primary teeth [15]. 

The technique is aimed to increase child’s compliance and operator comfort as local anesthesia is eliminated. In addition to caries sealing, it is expected that a child will have a less traumatic dental experience in his early life and he will probably return for more difficult treatment in the future [16]. 

It is well-known that, the pulp-to-crown ratio in primary posterior teeth is larger than the ratio in permanent molars, leading to a reduced degree of pulpal protection [17]. Therefore, selective caries removal, such as indirect pulp capping, leaving a layer of carious dentine near the pulp under the restoration, or leaving carious dentine under the crown (the Hall technique), help to preserve the vital pulp [13,18,19]. Until this point, the use of Hall technique for carious primary molars remains controversial.

The aim of this review was to provide an updated search on the Hall technique description, indication, contraindication, advantages, concerns, success and failure, cost-effectiveness, acceptability, and preference in pediatric dentistry, and to compare the Hall technique with traditional crown preparation and conventional treatment options for carious primary molars.

## 2. Materials and Methods

Electronic databases, “Cochrane Database”, “PubMed”, “Science Direct”, and “Google Scholar”, were searched to identify relevant studies published in the period from 1991 to 2018. Abstracts and full texts were explored to identify studies that described the Hall technique and its indication, contraindication, advantages, disadvantages, success, and failure in pediatric dentistry. Articles that studied the acceptance and preference of Hall technique by dentists, parents, and children were included in the review. In addition, studies that compared the Hall technique with traditional crown preparation and conventional restoration were also included. A combination of the key words; Hall technique, use, primary molars, pediatric dentistry; were used in the search. 

## 3. Results and Discussion

### 3.1. Description of the Hall Technique

The Hall technique does not require local anesthesia, caries excavation, or tooth preparation. At the beginning, orthodontic separators should be placed between the contact point of the primary molar using two pieces of dental floss [20] or the elastic separating pliers [21]. The separators are left in place for five days. Then, after separators removal, the size of the PMC that is tight enough to give a feeling of ‘spring back’ during seating should be selected. Then, a glass ionomer luting cement (GIC) (Type I) is placed inside the crown, and the crown is pressed tightly until it is fully seated on the tooth. After that, the excess cement must be removed quickly. The patient is then asked to maintain biting on the crown for two minutes till the cement is completely set and the crown is fully seated in position. Finally, the remaining cement must be removed, the contact areas flossed, and the child is discharged. The child must be advised that the feeling of the crown being high during biting will be resolved within one or two days [20]. Regarding the use of orthodontic separators before placement of Hall PMCs, a Chi square analysis revealed no relationship between the use of separators and the adequate Hall crowns fit (*p* = 0.810) [22].

There was no considerable difference in the time taken to discuss the procedure and complete it between PMCs placed by Hall technique and other restorations when they were done at a single visit. The mean time to discuss and finish the restoration was almost 11 min (range from 4 to 32 min) for the traditional restorations. However, the average time was around 12 min (range from 2 to 40 min) for the Hall crowns. For 64% of the patients, the treatments were done at one appointment and for 48% of the patients, the treatment was accomplished at separate appointments. For the 13% of the PMCs placed by Hall technique, orthodontic separators were applied, which demanded an additional visit. GDPs had a good experience in placing most of the control restorations, which explains the reduced working time [22].

### 3.2. Indications and Contraindications of the Hall Technique

Not every child, carious primary molar in that child, or dentist is suitable for the Hall technique. In addition, application of the Hall technique without a comprehensive prevention program is unlikely to reach the goal of most pediatric dentists, which is, having carious primary teeth that exfoliate with the minimum risk of infection or pain [20]. Indications and contraindications for the use of the Hall technique are listed in Table 1.

### 3.3. Advantages of the Hall Technique

The Hall technique is acceptable and preferable to many children, parents, and dentists. It has many advantages that are summarized in Table 2.

### 3.4. Concerns of the Hall Technique

The Hall technique has some concerns regarding its use. The technique is time consuming, as orthodontic separators are needed, which indicates an additional visit. It does not include occlusal preparation before cementation of the crown. This could lead to premature contacts after crown cementation and increase occlusal vertical dimensions (OVDs). However, adequate occlusal contacts are re-established at the recall visit after 1 year [23]. Furthermore, children need to withstand biting a rigid metal crown into its place, across relatively tight contact points, with no local anesthesia [14]. Moreover, metal crowns are not cosmetically acceptable to the child or the parents [12,27].

Because PMCs are fitted without occlusal tooth preparation, the occlusion might be temporarily opened. However, according to Innes et al., the occlusion would be adequately balanced by the next recall and no patient demonstrated tempo-mandibular-joint (TMJ) problems [14]. However, it was suggested that the sequela of premature unilateral contact requires more investigation with long-term follow-up periods via prospective randomized clinical trials [14].

**Figure 1:**
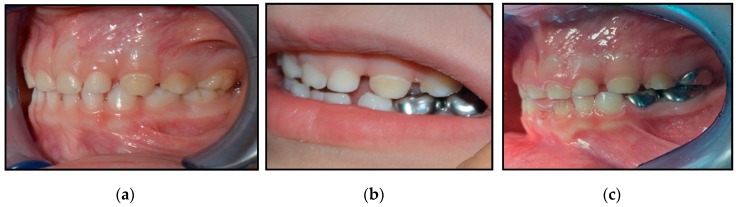
A four-year-old boy treated with Hall preformed metal crowns (PMCs) showing: (**a**) A pre-treatment lateral photograph showing the patient’s occlusion, (**b**) a post-treatment lateral photograph showing the patient’s occlusion (taken immediately after cementation of the last PMC), and (**c**) a one-year-recall photograph showing correction of the OVD.

A prospective clinical trial by Innes et al. in a general dental practice also confirmed that the Hall technique would be correlated with premature contacts after the cementation of crowns and elevations in occlusal vertical dimensions (OVDs). Using the Hall technique on second primary molars can cause slightly more of an increase in OVDs compared to that on first primary molar. Results showed that at one-year-recall, even occlusal contacts were re-established in all cases [22]. Supporting the previous results, the following images for a patient, which were taken by one of the authors (DHA) of this study, demonstrates the use of the Hall Technique for managing carious primary molars of an extremely uncooperative four-year-old boy. At approximately one-year-follow up, the OVDs had been corrected, as shown in Figure 1. Parent consent was taken before taking these photos.

According to Innes et al., the occlusion equilibrates rapidly, usually within few weeks [14]. Though it is better to follow up the child two weeks following placement of the crown to evaluate the occlusion, it was not possible due to the limitations of the general dental practice setting. Forming a perfect research design within the general dental practice environment was considered one of the research challenges [22]. 

It has been shown that there were no children who re-attended the clinic after using Hall technique with signs of occlusal problems and TMJ dysfunctions, or difficulty during eating at one or two-year recall visits [22]. In addition, OVDs were spontaneously corrected after almost 30 days following placement of crowns by the Hall technique [28]. 

Orthodontists regularly treat patients with bite planes anteriorly or posteriorly, which significantly increase the OVDs compared to Hall crowns. These appliances do not increase the risk of TMJ disorders when placed in a healthy child [29]. They temporary changes the vertical dento-alveolar growth, by a transient acceleration in lower facial height and fast eruption in the remaining teeth to reach even occlusal contacts for adaptation. The acceleration in lower facial height is adjusted by slowing in the vertical facial growth [30]. PMC placed by the Hall technique is acting in a way resembling the orthodontic appliance and no unfortunate outcomes were seen. A literature review by Luther in 2007 demonstrated no evidence to support the principle that premature occlusal interferences could cause temporomandibular joint (TMJ) dysfunction syndrome [31]. Although evidence is not conclusive whether HT may cause malocclusions, the risk cannot be discarded.

### 3.5. Hall Technique Versus Traditional Crown Preparation

#### Success Versus Failure

A retrospective study by Ludwig et al. in 2014 assessed the radiographic and clinical success of SSCs placed on primary molars using both the Hall technique and traditional crown technique which involves excavation of the whole carious lesion and tooth preparation before placement of SSCs over a period of five years [24]. The patients were followed up for at least six months or until failure; whichever came first. The success of crowns was graded based on Innes et al.’s criteria [14]. After an average of 15 months follow-up, 97% of crowns placed by the Hall technique were successful, while 94% of those placed by the traditional crown preparation were successful after a mean observation time of 53 months. Only two Hall technique cases showed failures of the Hall technique. Both failures were due to abscesses, with one crown causing symptoms after 5 months and the other was identified during routine dental examination after 11 months. None of the successful crowns caused symptoms of pain as reported in the dental record and follow up examinations were not needed to evaluate symptoms. Seven traditional SSCs failed. Five of them failed due to infection or abscess at an average time of 17 months. Furthermore, two SSCs failed due to unretained crown at four and 55 months, and when re-cementation of the SSCs of both teeth were successfully done. Of the successful traditional SSCs, one patient reported postoperative pain which did not require any further treatment. The results of this study were inconclusive due to certain limitations. First, it is a retrospective study, and the traditional crowns group was not an ideal control because most of these restorations were done earlier compared to the Hall technique group, and the patients were not randomly allocated to the treatment groups. Secondly, the study measured the success of the crowns in relation to the pulpal or caries conditions and at different performance times between the two techniques. However, it did not relate periodontal health or occlusion problems. Finally, a statistically significant relationship could not be found due to the less frequent failure incidence relative to the sample size [24]. 

Supporting the results of Ludwig et al. [24], Elamin et al. in 2019 found that the survival rate of both the Hall technique and the traditional crown technique were high (above 90%) after two years, with no statistically significant difference. The survival rate after one year was also high for both techniques—it was 94.5% for the Hall technique and 96% for traditional crown technique. There were 2.7% minor failures and 6.4% major failures in the Hall technique. However, 5.8% minor failure and 5.8% major failure were seen in the traditional crown technique. Minor failures included perforation or dislodgement of the SSCs without pain. Conversely, major failures include teeth that with pain that needed pulp therapy or extraction [32]. From the previous studies, we can infer that PMCs placed using the Hall technique or conventional crown technique have excellent survival rate.

### 3.6. Hall Technique Versus Other Restorations or Non-Restorative Approach

#### 3.6.1. Success Versus Failure

The similarity in the survival rates of many traditional restorations and crowns placed by the Hall technique is worthy of special attention, as in the Hall technique, there is no even partial excavation of caries before the placement of Hall crowns. The caries are being sealed under the crowns. This is considered as indirect evidence to support the Hall technique for decayed primary molars from some studies that examined the success of partial caries excavation and subsequent placement of a restoration, which stated that caries advancement was stopped or at least greatly caught up [33,34,35,36].

The need for alternative treatment modalities for decayed primary molars, such as the Hall technique or a prevention-only approach, might be questioned. This is due to the available conventional options with proven efficacy which are parts of standard teaching in all dental collages. Nevertheless, these techniques necessitate an early diagnosis by radiographs, followed by conventional restorations using local anesthesia and high-speed hand pieces which are not always applied in the primary care setting. Ideally, every child must have access to restorative and preventive care of great quality, but until that time arrives, there is still a need for alternative techniques that are easier and more acceptable to dentists, children, and their parents [14]. 

A split mouth randomized controlled clinical trial studied the clinical effectiveness of conventional restorations (control) placed in decayed primary molars, and crowns placed by Hall technique on the contralateral molars (matched radiographically and clinically) by GDPs, with a minimum recall period of 23 months. Conventional restorations that were used are: Amalgam, composite, compomer, glass ionomer, and fissure sealant. Success or failure of the used restorations and crowns were assessed using the usual clinical criteria, as listed in Table 3 [22]. 

The results of the above clinical trial showed that the success rates of the PMCs placed by the Hall technique were greater than the control restorations. Criteria of major failures were seen in 15% of control restorations. However, they were seen in only 2% of Hall crowns (*p* < 0.000). Furthermore, criteria of minor failures were noticed in 46% of control restorations but only in 5% of Hall crowns (*p* < 0.000). Pain from the restored tooth was demonstrated in 11% of control restorations, while it was seen in 2% of Hall crowns (*p* = 0.003) [22]. 

A retrospective study over a thirteen-year-period had evaluated crowns placed by the Hall technique. Crowns were considered successful if they were present until the last examination date before the child was lost to follow-up, or if the study cut-off date was reached and the crown still not lost or extracted. Furthermore, crowns were considered successful if the tooth shed normally. It was assumed that the shedding time of the first primary molars is at 10.0 years of age, and the shedding time of second primary molars is at 11.0 years of age. However, failure of crowns placed by the Hall technique was considered when the crowns were lost before shedding. Failure was also ascribed when the crowned teeth were symptomatic and required extraction. Teeth that were extracted due to orthodontic reasons but were not recorded at that time were all treated as failures [14]. Regarding the success rate of PMCs crowns placed by the Hall technique, 42% of the crowns were present until the last examination date before the child was lost to follow up, or until the cut-off date of the study. In addition, 34% of the crowns shed in their expected normal time. However, only 13% of the crowns were de-cemented and 11% were extracted because they became symptomatic, or for orthodontic reasons [14]. For all primary first and second molars, the survival rates of PMC placed using the Hall technique were around 73.4% after three years and 67.6% after five years. The data showed no significant difference in the survival rates of PMC placed using Hall technique on second primary molars compared to first primary molars after five years or following three years [14]. 

When comparing the results of Innes et al. [14] to the results of the systematic review published by Chadwick et al. [37] on restorations longevity in primary teeth, the success of Hall crowns (73.4% after three years and 67.6% after five years) [14] seems to be higher than the success seen in glass ionomer restorations (approximately 65% after three years and 32% after five years) and almost comparable to the success seen in composite restorations (approximately 78% after three years) [37]. However, the simplicity and the superiority of the Hall technique against regular composite should be taken into consideration. Nevertheless, care should be taken during comparison of the results of restorations’ longevity done in different clinical trials.

Another split-mouth, randomized control trial was conducted by Innes et al. in 2011 [13] as a continuation of their previous study in 2007 [22] but the recalls were done annually for five years (60 months) and at emergency appointments. Ninety-one patients out of 132 had 48-month minimum follow-up or both teeth had reached an endpoint (exfoliated, extracted, or censored). The principal result of this study was that Hall crowns were significantly more successful as long-term restorations compared to standard restorations placed by GDPs. The results showed that leaving caries and sealing it by Hall crowns were statistically significantly exceed GDPs’ standard restorations. They found that 92% of the Hall technique group and 52% of the control arm were successful according to the criteria described by Innes et al. in Table 4 [22]. None of the Hall technique teeth faced both ‘minor’ and ‘major’ failures. Eight teeth from the control group experienced ‘minor’ failures initially and consequent ‘major’ failures, but only one tooth in the control group demonstrated a ‘major’ failure (treated by pulp therapy or had an abscess) and an additional ‘minor’ failure (loss of the restorations) [13]. 

Regarding the minor and major failures of the Hall technique versus amalgam, composite, compomer, glass ionomer, and fissure sealant, 5% minor failure was seen in the Hall technique group and 42% minor failure was noticed in the control restorations after a minimum recall period of 48 months. The first minor failures occurred after 5 to 36 months. Categories of failures for the Hall technique were crown attrition (*n* = 1), loss of the crown (*n* = 1), impacted permanent first molars due to separators placement (*n* = 1), and caries at the margins of the crown (*n* = 1). However, failures in the control restorations were fracture/wear of the restorations (*n* = 3), loss of restorations (*n* = 21), radiographic progression of caries (*n* = 1), and secondary caries (*n* = 13). There was a statistically significant higher minor failure in control restorations in comparison to the Hall technique (*p* < 0.000001). Major failures were 3% in the Hall crowns group, with failures at 3, 17, and 31 months. However, major failures were 16.5% in control group. The first failures occurred after 1 to 60 months. Categories of failures for the Hall crowns group were either irreversible pulpitis (*n* = 1) or abscess (*n* = 2). However, major failures in control restoration were due to abscess (*n* = 12), broken and non-restorable teeth (*n* = 1), or irreversible pulpitis (*n* = 2). There was a statistically significant higher rate of major failures in control restorations compared with a Hall technique PMCs (*p* < 0.000488) [13]. 

The mean age of the included children in the study at 2007 was almost 7 years, teeth (with the Hall technique and control restoration) were exfoliated in 48% of the patients who reached an end point, though time to extraction or exfoliation was not reported [22].

In 2011, the results of Innes et al. were surprising [13]. Meanwhile, there was increasing evidence that supported sealing caries approaches (indirect pulp capping; partial caries excavation, and stepwise caries excavation), and all these techniques comprise some caries excavation and therefore removal of the superficial layer of plaque biofilm. Using the Hall crowns technique, the superficial layer of plaque, which is the most important layer in the biofilm for caries progression [15], was kept and sealed along with the carious lesion. These data suggest that the technique may stop caries advancement, or at least slows it to a degree that does not cause any concern in controlling caries in primary teeth. 

Hall crowns help GDPs in obtaining a durable and an effective seal in the crowded primary care settings. A realistic clarification for the low standard performance of restorations placed by GDPs was the major use of glass ionomer. Recently, there is good evidence that glass ionomer in not an ideal option for restoring multi-surface carious lesions [38]. Another description for the low standard performance of GDPs restorations was that no pulp therapies were performed at the first appointment for any one of the control teeth, although several primary molars had advanced carious lesions. Cavity preparations on progressively decayed teeth may produce a stressful pulp subsequently. Further placement of restorations with inadequate seal could worsen the tooth condition leading to ‘major’ failures. Nevertheless, since the lesions were similar in the extent and site, success of the Hall crowns indicate slowing and likely cessation of the caries activity and its progression [22]. 

Another longitudinal randomized clinical trial study supported the previous results of a higher success rate of PMCs placed by the Hall technique compared to other treatment approaches for cavitated primary posterior teeth [39]. This study included a younger children’s age than previous studies [13,22]. The mean age of children was 5.5 years. The treatment options were: Complete caries excavation and compomer restorations placement (CR); PMCs using the Hall technique (HT); and non-restorative caries treatment approach (NRCT) with opening the cavity and fluoride varnish placement. [39]. Results showed minor failure in 20 cases from the 148 cases: CR = 7%, HT = 1%, NRCT = 5%, (*p* = 0.002). Significant differences were found between NRCT and HT groups (*p* = 0.030) and between HT and CR groups (*p* = 0.011) with the HT showing better results. Causes for failure in the NRCT group were caries advancement or reversible pulpitis not requiring pulpotomy. However, failure reasons in CR group were restorations fracture, restorations loss, or secondary caries. In the HT group, only one case demonstrated new caries lesion around the crown margin after one year [39].

Regarding the major failure, nine out of 148 teeth expressed major failures (CR = 5, NRCT = 4). However, no major failure was seen in the HT group (*p* = 0.002). In the NRCT group, reasons for failures were the presence of abscesses or signs of irreversible pulpitis. In the CR group, reasons for failures were the development of abscess or reversible pulpitis which required pulpotomy. Results showed that success or failure of the restorations was not significantly related to the dentist’s experience (*p* = 0.13) [39]. 

Regarding the non-restorative caries treatment, Silver diamine fluoride (SDF) is considered a medical non-invasive treatment option for carious primary molars. SDF is a combination of an antimicrobial (25% Ag), a remineralizing agent (5% F), and a stabilizing antiseptic agent (8% ammonia). It is a brush-on liquid which can arrest 81% of carious lesions [6]. SDF attains the goal of sealing carious lesions without removal of the carious material similar to the Hall crown technique. However, it causes unfavorable black staining on the arrested carious lesions which mostly concerns the clinicians not the parents [40]. Table 4 summarizes the findings of several studies that calculated the success rates of different caries management approaches.

#### 3.6.2. Cost-Effectiveness

Regarding the cost-effectiveness of different decayed primary molars treatment strategies, a study compared the cost-effectiveness of conventional restoration and Hall technique. All primary posterior teeth had cavitated lesions with asymptomatic vital pulps. The carious molars in 5-year-old children were followed till shedding of the teeth to study the success of the different strategies [26]. Cost-effectiveness was assessed using a public-payer perspective within the German healthcare system. Costs in Euros per year of a tooth retention were calculated. The study followed the guidelines for economic health analyses by Husereau et al. [41]. This analysis assessed costs with different evaluated health outcomes. The results showed that conventional restorations were less successful and more expensive than the Hall technique [26]. 

In 2018, a study by Schwendicke et al. compared the cost-effectiveness of HT crowns with conventional restorations in a five-year randomized clinical trial in Scotland. Initial and retreatment costs involving endodontic treatment, restorations, and extractions were calculated. They found that teeth with HT crowns had significantly higher survival rate compared to teeth treated with conventional restorations. In addition, the percentage of teeth retained without pain or need for either extraction or endodontic treatment was significantly greater in HT. The initial costs of HT crowns were higher than those of the conventional restorations. However, when adding the retreatment costs, HT crowns were found to be less expensive. These results indicate that HT is considered more cost-effective than conventional restorations [25].

Schwendicke et al. published another randomized controlled clinical trial in 2018 that supports the cost-effectiveness of the Hall technique compared to other treatment options for carious primary molars. The study compared the cost-effectiveness of HT, NRCC, and conventional restorations in Germany. NRCC involves the removal of overhanging enamel and dentin followed by regular removal of biofilm and application of fluoride. The results revealed teeth treated with HT demonstrated greater survival rate compared to teeth treated with NRCC and conventional restorations. In addition, HT costs were less than conventional restorations and NRCC. Thus, HT had superior cost-effectiveness than both conventional restorations and NRCC [42]. 

Although SDF shares similar indications and contraindications as the Hall technique, since the survival rates of SDF and Hal technique are quite similar, it was considered as a highly cost-effective technique to treat caries lesions [43]. The results of the previous studies approve that the Hall technique is the most cost-effective caries treatment approach.

### 3.7. Preference and Acceptability of the Hall Technique

In children, treating decayed primary molars involving many surfaces is challenging. Compared to treatments in adults, pediatric dentists should take into consideration some factors such as patient’s cognitive development, age, treatment option, and pain perception. These factors have fundamental roles in the choice of dental treatment [44,45]. A study compared pain perception and children’s behavior throughout the procedure and techniques’ tolerability to dentists and parents. Using Frankl scale, patients demonstrated less negative behavior after treatment with either HT or NRCT compared to children treated by CR. However, patients’ pain perception was similar in all treatment groups. In addition, the three treatment options were similar in parent’s acceptability. Dentists rated HT and NRCT as being ‘easy’ or ‘very easy’ compared to CR [46].

Another study showed that 81% of dentists, 83% of caregivers, and 77% of patients preferred the Hall technique. The use of separators did not affect the preference for the procedure, the discomfort or pain levels in the child, as assessed by his dentist, after cementation of the crown from the perspective of the child, his parents, or the dentist. When the Hall crowns and control restorations were demonstrated at one appointment, the predilections was not reliant on which kind of treatment was done first (*p* = 0.203) [22]. 

On the other hand, aesthetic problems related to PMCs placed by the Hall technique can be a concern of patients and their parents compared to conventional aesthetic restorations. Some studies reported that one of the reasons specified by dental practitioners for not using stainless steel crowns for multi-surface carious lesions, extensive dental caries, and after pulpal treatment, was because PMCs are not aesthetically acceptable to the parent or child, although most dentists recognized that crowns are the most durable restoration for primary molar teeth [12,27]. Although some parents complained from the aesthetics of metal crowns, once the dentist explained all the advantages to the parents, they agreed with the treatment [12]. 

Some parents may complain from the appearance of PMCs [47,48]. Aesthetic crowns are of raising interest in the treatment of carious primary posterior teeth but are not commonly applied due to the need of tooth preparation, failure to place them by Hall technique and the increasing susceptibility to fracture [47].

The use and preference of Hall technique as one of the treatment options for primary posterior teeth were assessed in Scottish general dental practice. In addition, the awareness and predilections for methods of more future training for dentists who were still not using the technique were also assessed. A high percentage of respondents who treated children (*n* = 665) stated that they had heard about Hall technique, and 48% indicated that they were using the technique (*n* = 318). The frequency of using the technique varied from ‘very frequently’ (*n* = 25) to ‘never’ (*n* = 340). For general practitioners who had never used the technique, a lack of knowledge and confidence were the main barriers against its use (26%). A preference for alternative treatment (8%) and lack of stock materials (8%) were other barriers [49]. 

Treatment preference was evaluated by a questionnaire among paediatric dentistry postgraduates for the treatment of children with different caries severity in a primary molar tooth [50]. A wide range of treatment options were chosen for non-anxious patients with no indication of pulpal treatment, while the Hall technique was preferred by 16 of 32 students for anxious patients. As such, the Hall technique was the preferred treatment option by half of the postgraduate students for asymptomatic carious primary molars in anxious children [50].

In conclusion, the Hall technique is more acceptable and preferable non-invasive caries treatment option by parents and dentists than the more invasive caries treatment options. Although no study compared the preference and acceptability of Hall technique and SDF, a high level of acceptance of the stain caused by SDF was noticed. Like the Hall technique, most of the parents preferred SDF instead of other invasive caries treatment options. The majority of parents preferred black stains and unknown outcome over the injection, drill, long treatment time, and pain. The acceptability increased when their child required advanced methods of behaviour management [51]. 

Further longitudinal studies are needed to study the effectiveness and success rate of the combination of different non-invasive caries arresting approaches. A study on the effect of using SDF with the Hall technique is suggested. In addition, the effectiveness of combination of the Hall technique, SDF, and fluoride varnish need further investigations.

## 4. Conclusions

The Hall technique can be an effective addition to the clinician’s range of treatment options for the carious primary molar. It is strongly supported that crowns placed by the Hall Technique treatment option have promising results, showing high acceptability and longevity; and low failure rate for managing carious primary molars compared to conventional treatment options commonly applied in primary care settings. In addition, the survival of SSCs is high, whether provided using the Hall technique or traditional preparation by a pediatric dentist. The Hall technique should be chosen in some restricted cases. If SSCs can be placed using traditional crown preparation, dentists need to do that instead of the Hall technique due to some disadvantages of the Hall technique.

## Figures and Tables

**Table 1:** Hall technique indications and contraindications for carious primary molars.

Indications of the Hall Technique
Teeth with occlusal caries, non-cavitated, if the patient is unable to accept fissure sealant, partial caries removal or conventional restoration [20]. Teeth with proximal caries either cavitated or non-cavitated if the patient is unable to accept partial caries removal, or conventional restoration [20]. Hall technique is mostly indicated to be used in routine general dental practice [20].
Contraindications of the Hall Technique
Tooth with signs or symptoms of dental infection or irreversible pulpitis [20]. Crowns severely destructed with caries, which considered non-restorable [20]. Very young children who do not understand the procedure or tolerate biting the crown into its position without local anesthesia [14,23].

**Table 2:** Advantages of the Hall Technique for managing carious primary molars.

Advantages of the Hall Technique
It is a non-invasive procedure in which the crown is cemented without local anesthesia, caries excavation, or tooth preparation [23]. It is a quick procedure that limits child’s anxiety [24]. It is considered as a less traumatic technique for the child [23]. It seals in carious lesion and could arrest caries or at least slow it down [15]. It improves pulpal health [23]. It increases the access to dental care, decrease percentages of untreated dental caries and deliver a restoration that will permit natural tooth exfoliation [24]. It is more cost-effective than conventional restorations [25,26]. If done at a single visit, the time needed to complete the procedure is minimal [22].

**Table 3:** Success and failure criteria of conventional restorations and the Hall technique crowns according to Innes et al. [22].

	Criteria
**Success**	Restorations or crowns appear satisfactory and no interventions were required. No clinical or radiographic signs of any pulp disease. Normal tooth exfoliation.
**Minor Failure**	Secondary caries, or new caries radiographically or clinically. Restoration fracture or wear that requires intervention. Restoration or crown loss, while the tooth was considered restorable. Reversible pulpitis that does not require pulpotomy or extraction.
**Major Failure**	An abscess or an irreversible pulpitis indicating extraction or pulpotomy. An inter-radicular radiolucency or an internal root resorption. If the restoration or crown was lost, or the tooth was non-restorable.

**Table 4:** Success rate success rates of different caries management approaches.

Caries Management Approaches	Success Rate	Follow-Up
Hall Technique	94.5% [32]	1 year [32]
	97% [24]	15 months [24]
	73.4% [14]	3 years [14]
	94% [24]	53 months [24]
	67.6% [14]	5 years [14]
Traditional Crown Preparation	96% [32]	1 year [32]
Composite	78% [37]	3 years [37]
Glass Ionomer	65% [37]	3 years [37]
	32% [37]	5 years [37]

## Abbreviations

CR	complete caries excavation and compomer restoration
ECC	early childhood caries
GDPs	general dental practitioners
GIC	glass ionomer cement
HT	Hall technique
NRCC	non-restorative cavity control
NRCT	nonrestorative caries treatment
OVDs	occlusal vertical dimensions
PMCs	preformed metal crowns
SSCs	stainless steel crowns
TMJ	temporomandibular joint
